# Effectiveness of manual lymphatic drainage in intensive phase I therapy of breast cancer–related lymphedema—a retrospective analysis

**DOI:** 10.1007/s00520-023-08210-7

**Published:** 2023-12-05

**Authors:** Renato G. Kasseroller, Erich Brenner

**Affiliations:** 1 Bad Vigaun Medical Centre, Karl-Rödhammer-Weg 91, 5424 Bad Vigaun, Salzburg, Austria; 2 Institute for Clinical and Functional Anatomy, Medical University of Innsbruck, Müllerstrasse 59, 6020 Innsbruck, Austria

**Keywords:** Manual lymphatic drainage, Complex physical decongestive therapy, Breast cancer–related lymphedema

## Abstract

**Background:**

The standard therapy for lymphedema of any origin is complex physical decongestive therapy (CDT). It comprises manual lymph drainage (MLD), compression therapy (CT), exercise therapy (ET), skincare, and patient education. Additionally, intermittent pneumatic compression (IPC) can be applied. However, the contribution of MLD to decongestion is repeatedly questioned.

**Patients and methods:**

This study re-analyzes a previous study during a 3-week decongestion period, comparing two different types of compression bandaging at the weekend. Sixty-one patients with unilateral breast cancer–related lymphedema were included. The patients received the same therapy (CDT + IPC) except for the different weekend compression bandaging. MLD was performed twice a day on weekdays. The volume of the affected arm was measured on days 1, 5, 8, 12, 15, 19, and 22. For the analysis, the data of both study groups were pooled.

**Results:**

During the week, the patients showed a significant volume reduction (− 155.23 mL (week 1), − 101.02 mL (week 2), − 61.69 mL (week 3), respectively; *p* < 0.001 each) with a high effect size. On the weekends without MLD, they showed a slight, but also significant increase (12.08 mL (weekend 1), 8.36 mL (weekend 2), 4.33 mL (weekend 3), respectively; *p* < 0.001 each) with a medium effect size.

**Conclusions:**

We showed a strong effect of MLD on volume reduction. Differences from other studies are the larger study population and the more intensive application of MLD. If applied intensively, MLD is strongly decongestive during a 3-week decongestion therapy for breast cancer–related lymphedema.

**Supplementary Information:**

The online version contains supplementary material available at 10.1007/s00520-023-08210-7.

## Introduction

Complex decongestive therapy (CDT) is a comprehensive concept for lymphedema therapy [1]. CDT consists of several components that work together to improve lymph flow, reduce swelling, and relieve pressure on the affected region. The main components of CDT are:

1. Manual lymphatic drainage (MLD): MLD is a special massage technique stimulating the lymphatic system and promoting lymph flow. Specially trained therapists perform MLD and can help to remove congestion and reduce swelling.
2. Compression therapy (CT): Compression therapy uses compression bandages (CB) or compression garments (CG) to increase the pressure on the affected area. This promotes lymph flow and reduces swelling. CB or compression systems are often combined with MLD in the decongestion phase (phase I), while CG is usually worn in the maintenance phase of therapy (phase II).
3. Exercise therapy (ET): Targeted exercises and exercise therapy are important to activate the muscle pump and support lymph flow. The activities should be tailored to the patient’s individual needs and abilities and should be performed under the guidance of a physiotherapist or lymph therapist.
4. Skincare: Good skin care is important to reduce the risk of infections and skin problems, which are more common in people with lymphedema. The skin should be clean and well moisturized, and patients should observe for signs of infection or skin irritation.
5. Self-management and patient education: Patients should be trained to continue their therapy at home, including self-massage, applying compression bandages or systems, and performing exercises. Self-management is crucial for the long-term success of CDT.

CDT is individually tailored to the patient’s needs and usually requires close collaboration between the patient and the treatment team, which may include doctors, physiotherapists, lymphatic therapists, and other specialists. By combining these elements of therapy, CDT can help alleviate the symptoms of lymphedema and improve the quality of life of those affected.

Although MLD has been used in many countries for decades, recent systematic reviews, including a Cochrane systematic review with six randomized trials, could not demonstrate any additional benefit of MLD in the context of CDT [2, 3]. Recent studies also failed to demonstrate an additional benefit [4–8]. In addition, an immanent goal lies in the cost reduction of the therapy, which can be achieved by eliminating MLD with the necessary therapists.

In 2010, we published a study in which we examined 61 patients (December 2007 to May 2008) with unilateral breast cancer–related lymphedema (BCRL) during a 3-week inpatient decongestion phase (phase I) [9]. The aim of this study at that time was to compare the effect of an alginate dressing with that of a conventional dressing at weekends. No MLD and no ET were performed on the weekends. We could show that during the week, i.e., during CDT with MLD and ET, the volumes decreased, while at the weekends, i.e., without MLD and ET, the volumes did not decrease further but increased slightly. Since we had a different goal for this study, we documented these volume changes but did not analyze them further.

In this paper, we now want to make up for this and analyze the volume changes during the 3-week decongestion phase of CDT with MLD and ET (Monday to Friday) and without MLD and ET (Saturday and Sunday). Our basic assumption is that the observed volume reduction during the week (Monday to Friday) is mainly due to MLD, while the observed volume increase at the weekend (Saturday and Sunday) is due to the absence of MLD on these days.

## Material and method

The study was performed in accordance with the ethical standards as laid down in the 1964 Declaration of Helsinki and its later amendments. The Ethics Committee in Salzburg (Austria) approved the original study under number 888. All patients gave their informed consent to participate in the study.

The details of the recruitment, the study protocol, and the baseline data of the patients have already been presented in detail in the original publication [9].

Between December 2007 and May 2008, we recruited 61 patients with unilateral breast cancer–related lymphedema (BCRL) undergoing 3 weeks of inpatient lymphological rehabilitation (decongestion phase, phase I). The patients had lymphedema in the axillary tributary region after axillary dissection (level I or II) because of treatment of their breast cancer with modified mastectomy (five patients) or lumpectomy (56 patients). None of these patients showed additional alterations of the skin, such as infections, hyperkeratosis, papillomatosis, fistulae, cysts, or ulcerations. Thirty-five patients had radiation, three of them adjuvant chemotherapy. Forty-one patients underwent a non-standardized treatment with MLD and compression garment 6 months prior to this admission. Randomization took place before admission to the treatment by assigning the patients randomly to one of the two alphanumeric groups, with group (A, *n* = 31) receiving conventional bandaging, and group (B, *n* = 30) alginate bandaging. The mean age of the patients was 57.4 ± 8.926 years (maximum 81 years, minimum 28 years). All patients had unilateral lymphedema stage 2 (*n* = 43) or 3 (*n* = 18) according to ISL staging [10] for at least 6 months up to 5 years before inclusion (Table 1).

**Table 1:** Patients’ characteristics

		Group A conventional bandaging	Group B alginate bandaging	Total	Significance
Age [yrs]		59.06 ± 7.76	55.60 ± 9.86	57.36 ± 8.95	*p* = 0.132
BMI [kg/m^2^]		27.07 ± 3.00	25.09 ± 2.78	26.19 ± 3.01	*p* = 0.022
ISL stage [*n*]	2	22	21	43	*χ*^2^ = 0.934
	3	9	9	18	
Volume difference of the affected arm [*n*]	+ 20–25%	17	16	23	*χ*^2^ = 0.879
	+ 25–40%	11	12	23	
	+ > 40%	3	2	5	

All patients were treated with CDT as phase I (decongestion phase) during their stay. All patients received MLD twice a day from Monday to Friday, lasting 90–120 min. MLD was performed on all patients by a single therapist who had received her training at the Dr. Vodder Academy in Walchsee, Austria. A compression bandage was applied during the treatment, including the fingers. Textile-elastic bandages of one single manufacturer (Rossidal KS®, Lohmann and Rauscher, Vienna) were used. The supportive lining was done with cottonwool bandages and foam pads that were positioned individually. The fingers were bandaged in a conventional way with a gauze bandage, usually, two 6 cm [2.5"] wide bandages and one 4 cm [1.5"] wide bandage are enough in the finger region. The short stretch was applied from hand position B to proximal arm position G' in eight tours, overlapping (from distal to proximal and back and again to proximal). For the hand, one usually needs one 6 cm [2.5"] wide short-stretch bandage; for the rest of the arm, one 8 cm [3.0"] wide and two 10 cm [4.0"] wide bandages. A complete underlayer cottonwool was applied. The pressure under bandaging was 30 mmHg (4 kPa) distally, decreasing proximal, corresponding to compression class II (RAL). Patients were instructed not to remove the CB before going to sleep (approximately 9 p.m.).

A balm containing polidocanol was applied evenly for skin care. As additional exercise therapy (ET), a uniform water gymnastics program was performed in the morning and dry gymnastics in the afternoon. An accompanying intermittent pneumatic compression (IPC) was applied daily from the third day of treatment and on Saturdays and Sundays for 30 min each with an initial pressure of 5.3 kPa (40 mmHg; Lympha Press®, Villa Sana, Weiboldshausen, Germany), as recommended by the manufacturer. In the original study, the patients were divided into two groups for the weekend. One group (A) received a conventional short-stretch bandage for the weekend, and the second group (B) a semi-rigid alginate bandage (Alegro Alginate, Alegro Medical, Homburg, BRD), a low-stretch bandage with an average elasticity of 55% impregnated with a calcium-alginate paste. The bandage with a high working pressure, but a low pressure at rest, was applied directly to the skin. Additionally, a dermato-protective effect was achieved by the alginate impregnation. The fingers were bandaged conventionally with a gauze bandage. The alginate bandage was applied in two crossed layers from the back of the hand to just below the axilla. The patients had the possibility to moisten the alginate bandage by absolute freedom of choice during the whole day. This bandaging was not removed until Monday morning.

The detailed treatment plan can be found in Table 2.

**Table 2:** Treatment plan for both study groups (day 22 omitted)

	MON	TUE	WED	THU	FRI	SAT	SUN
WEEK 1							
Day	01	02	03	04	05	06	07
Complex decongestive therapy	A + B	A + B	A + B	A + B	A + B		
Apparative intermittent compression			A + B	A + B	A + B	A + B	A + B
Structured exercise therapy	A + B	A + B	A + B	A + B	A + B		
Free exercise therapy						A + B	A + B
Conventional bandaging	A + B	A + B	A + B	A + B	A	A	A
Alginate bandaging					B	B	B
WEEK 2							
Day	08	09	10	11	12	13	14
Complex decongestive therapy	A + B	A + B	A + B	A + B	A + B		
Apparative intermittent compression	A + B	A + B	A + B	A + B	A + B	A + B	A + B
Structured exercise therapy	A + B	A + B	A + B	A + B	A + B		
Free exercise therapy						A + B	A + B
Conventional bandaging	A + B	A + B	A + B	A + B	A	A	A
Alginate bandaging					B	B	B
WEEK 3							
Day	15	16	17	18	19	20	21
Complex decongestive therapy	A + B	A + B	A + B	A + B	A + B		
Apparative intermittent compression	A + B	A + B	A + B	A + B	A + B	A + B	A + B
Structured exercise therapy	A + B	A + B	A + B	A + B	A + B		
Free exercise therapy						A + B	A + B
Conventional bandaging	A + B	A + B	A + B	A + B	A	A	A
Alginate bandaging					B	B	B

A: group A with conventional bandaging

B: group B with alginate bandaging

The volume of the affected arm was measured at seven time points (days 1, 5, 8, 12, 15, 19, 22) according to Kuhnke’s method [11].

For the present evaluation, we have combined both groups, as we want to investigate here whether MLD applied twice a day during the week has an effect in terms of volume reduction. The form of bandaging at the weekend does not play a role.

For statistical analysis, we performed exploratory data analysis and two-tailed paired *t*-tests for the volumes using GNU pspp 1.6.2-g78a33a (Free Software Foundation, Inc.). For the effect size measures, Cohen’s *d*_2_ was calculated for paired *t*-tests [12, 13].

## Results

The volumes of the diseased arm of all 61 patients from all examination days were available for evaluation (Table 3). There was a decrease in volume from Monday (day 1, 8, 15) to Friday (day 5, 12, 19) and a slight increase in volume from Friday (days 5, 12, 19) to Monday (days 8, 15, 22).

**Table 3:** Volumes of the diseased arms [mL]

*N* = 61	Mean value	SD	Min	Max	95% CI	*t*-tests	Cohen’s *d*_2_
Day 1	2999.79	553.51	1985	4222	[2858.03, 3141.55]	*t*(60) = 10.38, *p* < 0.001	1.329
Day 5	2844.58	526.21	1902	4080	[2709.79, 2979.33]		
						*t*(60) = − 5.45, *p* < 0.001	− 0.698
Day 8	2856.74	528.80	1900	4110	[2721.31, 2992.17]		
						*t*(60) = 8.36, *p* < 0.001	1.070
Day 12	2755.72	503.79	1810	3970	[2626.69, 2644.00]		
						*t*(60) = − 4.46, *p* < 0.001	− 0.571
Day 15	2764.08	503.70	1810	3950	[2635.00, 2893.09]		
						*t*(60) = 7.33, *p* < 0.001	0.939
Day 19	2702.39	489.42	1822	3822	[2577.05, 2827.74]		
						*t*(60) = − 3.90, *p* < 0.001	− 0.499
Day 22	2706.72	488.95	1825	3830	[2581.49, 2831.95]		

During the workweek 1, the volume decreased by 155.23 mL in average (5.17% of the initial volume), during workweek 2, the volume decreased by 101.02 mL (3.37% of the initial volume), and in workweek 3, the decrease amounted for 61.69 mL (2.06% of the initial volume). During the weekends, the volumes increased in average by 10.16 mL (weekend 1), 8.36 mL (weekend 2), and 4.33 mL (weekend 3). Overall, the volume of the affected arm decreased by 293.07 mL (difference D22–D01) or 9.77% of the initial volume.

The volume changes were significant in all cases. The effect sizes (Cohen’s *d*_2_) show—according to Cohen’s rules of thumb—in each case a large effect (|*d*|> 0.8) for the volume decreases during the working week and a medium effect (0.5 <|*d*|< 0.8) for the volume increases at the weekend.

## Discussion

This re-analysis of previously published data confirms that a strict therapy regime in a 3-week decongestion phase in patients with BCRL leads to a significant volume reduction of almost 10% of the initial volume.

The question, therefore, arises as to which component of CDT has led to the great decongestion effect here.

In this study, we supplemented the classical CDT with IPC, which was applied daily from the 3^rd^ day onwards, also at weekends. However, since there was also an increase in volume, albeit small, at the weekends, the decongestant effect during the working week cannot be attributed to the IPC. This conclusion is also strengthened by the fact that the IPC was only applied from the third day onwards in the first week, i.e., there were only three IPC applications in the first working week instead of the usual five, but the volume decrease was greatest in the first working week.

Self-management and patient education as an essential component of CDT are aimed at the maintenance phase (phase II) and therefore excluded as a factor for the decongestion phase.

Compression therapy cannot be the decisive factor here, either. During the working week, all patients received the same conventional compression bandage; only at the weekend did some patients receive an alginate bandage instead of the conventional bandage. Thus, compression therapy cannot be considered a significant factor for the observed volume decrease during the working week.

The skin care, in our case with a cream containing polidocanol, is to reduce the risk of infections and skin problems but does not influence the volume and possible increases or decreases.

Exercise therapy (ET) certainly has an influence. It activates the muscle pump and thus promotes lymph flow. The improved lymph flow, in turn, can reduce the volume of the diseased limb. In our case, physiotherapy during the working week consisted of a uniform water gymnastics program in the morning and dry gymnastics in the afternoon. At the weekend, there was no structured ET, but the patients activated their muscle pump through their own measures (independent visits to the swimming pool, walks, etc., in each case with compression), thus stimulating the lymph flow.

This leaves MLD as the last component of CDT, which can be responsible for the different volume changes. With our patients, we assume that MLD has caused the observed major effects. However, this is contrasted by studies that deny precisely this decongestive effect of MLD.

In their meta-analysis, Huang et al. compared, among others, six clinical studies that investigated the decongestant effect of MLD compared to “standard therapy” [2]. The weighted mean volume difference was 75.12 mL (95% CI: − 9.43, 159.58) in favor of MLD application. The test for the overall effect yielded a *Z* of 1.74 with a probability of *p* = 0.08. The maximum probability of falsely rejecting the null hypothesis—here: both therapies have the same effect—is thus 8%, i.e., insignificant according to common parlance. Nevertheless, a clear trend in favor of MLD is recognizable.

Ezzo et al. used the same studies for their Cochrane review as Huang et al. [3]. In contrast to Huang et al., however, Ezzo et al. divided these six studies again after a more detailed analysis of the therapy regimes. In general, the results are always in favor of MLD; significant, for example, are the results for the comparison of MLD with compression bandaging versus compression bandaging alone (2 studies) for the outcomes of volume reduction in mL and percentage volume reduction as well as for the comparison of MLD with compression stocking versus IPC with compression stocking.

In their meta-analysis, Liang et al. [14] included finally eight RCTs, some of them also part of the previously mentioned meta-analyses [2, 3]. They summarized that MLD cannot significantly reduce lymphedema in patients after breast cancer surgery.

Lin et al. [15] analyzed ten RCTs and summarized that pain of BCRL patients undergoing MLD is significantly improved, while their findings did not support the use of MLD in improving volumetric of lymphedema and quality of life.

Xing et al. [16] analyzed and summarized seven systematic reviews and meta-analyses. They did not recommend the addition of MLD to CDT [sic!] or compression therapy for patients with BCRL based on the results of the present review. More well-designed and large RCTs are needed to provide a higher level of evidence to confirm the role of MLD in CDT.

A common conclusion of all these meta-analyses was that well-designed RCTs with a larger sample size are required. An overview of the studies included in these previously described meta-analyses is given in Table 4. A further study, which was hitherto not included in a meta-analysis, was made by De Vries and colleagues [8]. They compared BCRL patients with three different therapy concepts over 3 weeks with 14 therapy sessions. One group (*n* = 64) received CDT with placebo MLD, a second group (*n* = 63) received classical CDT including classical MLD, while the third group (*n* = 63) received CDT with fluoroscopy-guided MLD. Unfortunately, the authors did not give absolute volumes but limited themselves to percentages. The excess lymphedema volume (volume sick—volume healthy) decreased after 3 weeks of intensive treatment in each group: 5.3 percentage points of percentage excess volume (corresponding to a relative reduction of 23.3%) in the fluoroscopy-guided MLD group, 5.2% (relative reduction of 20.9%) in the traditional MLD group, and 5.4% (relative reduction of 24.8%) in the placebo MLD group. However, no clinically significant differences in volume reduction were found between the groups. This raises the question of what effect the placebo MLD had. In this group, deep massage was performed with relaxing transverse movements of the ipsilateral neck, back, shoulder, arm, and hand muscles.

**Table 4:** Overview of systematic reviews and meta-analyses

Analyzed studies	Johansson et al. [18]	Johansson et al. [19]	Andersen et al. [20]	Sitzia et al. [21]	Williams et al. [22]	McNeely et al. [23]	Didem et al. [24]	Szolnoky et al. [25]	Devoogdt et al. [26]	Bergmann et al. [4]	Gradalski et al. [5]	Cho et al. [27]	Zhang et al. [28]	Devoogdt et al. [29]	Tambour et al. [6]	Gol and Aghamohamadi [30]	Sen et al. [7]
Huang et al. [2]	+	+	+	+	+	+	+	+									
Ezzo et al. [3]	+	+	+	+	+	+	-		-								
Liang et al. [14]	+	+	+	+	+	+				+					+		
Lin et al. [15]			+			+			+		+	+	+	+	+	+	+

Except for the study by de Vrieze et al., all other studies have small group sizes, so any trend favoring MLD is not (yet) statistically significant. De Vrieze et al. aimed to establish fluoroscopy-assisted MLD as a better alternative to classical MLD. To this end, however, they did not apply CDT without MLD in the actual control group but instead used a placebo treatment, which they assumed had no draining effect on the lymph vessels. However, the described deep massage influences the musculoskeletal system and can have similar effects to exercise therapy.

### Limitations of this study

This study is a pooled re-analysis of an initially randomized controlled trial with a very special study design. This sequential design with three intervention phases of 5 days each, and interrupted and followed by three control phases of 2 days each (see Table 2), has the advantage of an identical intervention and control group, but the disadvantage that it might be difficult to compare with real randomized controlled trials. This re-analysis pooled the data of two groups, which differed in the treatment in the now control phases. By pooling these groups, the number of investigated patients increased and resulted in a significant volume reduction during the intervention phases. The number of patients exceeds the number of patients in the intervention groups of most of the clinical trials published up to now. As the initial study was done in 2008 [published 2010 as reference 9], not all original data were still available. For example, we could not find the absolute volumes of the healthy arms any more, but only the relative volume increase of the affected arms, expressed by the V-class of the LVF-classification by one of us [17].

## Conclusion

The re-analysis of the data indicates a significant volume reduction in patients with BCRL during a 3-week decongestion phase with a strict therapy regime. To our opinion, this effect can be attributed mainly to MLD.

Various studies, including meta-analyses, randomized clinical trials, and comparative studies, have been analyzed, each with different therapy regimes, group sizes, and measured outcomes. These studies exhibit mixed results regarding the efficacy of MLD, and although there is a trend favoring MLD, it is not statistically significant in most cases, and results vary significantly across studies. Other components of CDT, such as IPC, self-management, patient education, CT, skin care, and ET, have been examined and are deemed inconclusive in attributing to the observed volume decrease. The study’s findings, juxtaposed with the existing literature, suggest that MLD has indeed a measurable positive effect in decongestion phase (phase I) of the therapy of BCR lymphedema. This should not be extended to both phases, as our data only support a positive effect for phase I.

### Supplementary Information

ESM 1 (XLSX 24.8 mb)

## Data Availability

Raw data are supplied as supplementary file.
